# A Theoretical Model for Release Dynamics of an Antifungal Agent Covalently Bonded to the Chitosan

**DOI:** 10.3390/molecules26072089

**Published:** 2021-04-06

**Authors:** Luminita Marin, Marcel Popa, Alexandru Anisiei, Stefan-Andrei Irimiciuc, Maricel Agop, Tudor-Cristian Petrescu, Decebal Vasincu, Loredana Himiniuc

**Affiliations:** 1 “Petru Poni” Institute of Macromolecular Chemistry, 41A Gr. Ghica Voda Street, 700487 Iasi, Romania; lmarin@icmpp.ro (L.M.); anisiei.alexandru@icmpp.ro (A.A.); 2 Department of Natural and Synthetic Polymers, “Gheorghe Asachi” Technical University of Iasi, 700050 Iasi, Romania; marpopa@ch.tuiasi.ro; 3 Academy of Romanian Scientists, 54 Splaiul Independentei, 050094 Bucharest, Romania; 4 National Institute for Laser, Plasma and Radiation Physics, 409 Atomistilor Street, 077125 Bucharest, Romania; 5 Department of Physics, “Gh. Asachi” Technical University of Iasi, 700050 Iasi, Romania; 6 Department of Structural Mechanics, “Gh. Asachi” Technical University of Iasi, 700050 Iasi, Romania; tudor.petrescu@tuiasi.ro; 7 Department of Biophysics, Faculty of Dental Medicine, “Grigore T. Popa” University of Medicine and Pharmacy, 16 University Str., 700115 Iasi, Romania; decebal.vasincu@umfiasi.ro; 8 Department of Obstetrics and Gynecology, “Grigore T. Popa” University of Medicine and Pharmacy Iasi, 16 Universitatii Str., 700115 Iasi, Romania; loredanahiminiuc@gmail.com

**Keywords:** mathematical model, chitosan, bioactive aldehyde release, Scale Relativity Theory, multifractal, Riccati gauge, joint invariant function, SL(2R)-type group

## Abstract

The aim of the study was to create a mathematical model useful for monitoring the release of bioactive aldehydes covalently bonded to the chitosan by reversible imine linkage, considered as a polymer–drug system. For this purpose, two hydrogels were prepared by the acid condensation reaction of chitosan with the antifungal 2-formyl-phenyl-boronic acid and their particularities; influencing the release of the antifungal aldehyde by shifting the imination equilibrium to the reagents was considered, i.e., the supramolecular nature of the hydrogels was highlighted by polarized light microscopy, while scanning electron microscopy showed their microporous morphology. Furthermore, the in vitro fungicidal activity was investigated on two fungal strains and the in vitro release curves of the antifungal aldehyde triggered by the pH stimulus were drawn. The theoretical model was developed starting from the hypothesis that the imine-chitosan system, both structurally and functionally, can be assimilated, from a mathematical point of view, with a multifractal object, and its dynamics were analyzed in the framework of the Scale Relativity Theory. Thus, through Riccati-type gauges, two synchronous dynamics, one in the scale space, associated with the fungicidal activity, and the other in the usual space, associated with the antifungal aldehyde release, become operational. Their synchronicity, reducible to the isomorphism of two SL(2R)-type groups, implies, by means of its joint invariant functions, bioactive aldehyde compound release dynamics in the form of “kink–antikink pairs” dynamics of a multifractal type. Finally, the theoretical model was validated through the experimental data.

## 1. Introduction

Chitosan is a polysaccharide extensively investigated over the last decades due to its outstanding biologic properties, which make it an excellent candidate for the development of a large variety of biomaterials [1]. However, the intrinsic properties of chitosan are not sufficiently explored, in order to assure curing ability, and they should be improved by further chemical modifications [2,3]. Among various modification routes, the acid condensation reaction of chitosan with various aldehydes demonstrated efficiency towards a large realm of materials such as films, hydrogels and nanoparticles, in view of application in domains of contemporary interest, such as tissue engineering [4], drug delivery [5], soil conditioners [6], cosmetics [7], adsorption of carbon dioxide [8] or other hazardous pollutants [9], heavy metal sensing [10] and so on. However, recent studies demonstrated that the imination reaction of chitosan has a reversibility degree favored by the acidic medium in which chitosan is soluble [3]. This finding is in line with well-documented investigations that demonstrated that the imine linkage has a reversible character that proved beneficial for the construction of dynamic materials with an ability to adapt under the influence of various environment stimuli, such as moisture, temperature, pressure or pH [11,12,13]. These recent discoveries opened new perspectives for the use of chitosan as a matrix for the controlled release of the reversible bonded aldehydes [3,14]. Under the influence of environmental stimuli, the imination equilibrium is shifted to the reagents releasing the aldehyde, and the aldehyde consumption further triggers the equilibrium shifting towards the reagents and consequently towards the delivery of new aldehyde amounts. This simple mechanism becomes of particular importance when the released aldehyde has bioactive properties and its delivery is controlled by its consumption.

Usually, the models used to describe drug delivery dynamics are based on a combination of basic theories, derived especially from physics and computer simulations [15,16,17,18]. In such a conjecture, their description implies both computational simulations based on specific algorithms [18], as well as developments on standard theories. A class of models was developed on spaces with integer dimension—i.e., differentiable models, such as the Zero order model, First order model, Higuchi model, Hixson–Crowell model, Korsmeyer–Peppas model, etc. [19]. Another class of models was developed on spaces with non-integer dimensions and is explicitly written through fractional derivatives [20,21]—i.e., non-differentiable models, with examples including the fractal models [22]. Expanding on the last class of models, new developments have been made, based on Scale Relativity Theory, either in the monofractal dynamics, as in the case of Nottale [23], or in the multifractal dynamics, as in the case of the Multifractal Theory of Motion [24,25]. Our group has recently published in this framework, proving a good match for describing various drug delivery systems [26,27].

Both in the context of Scale Relativity Theory in the sense of Nottale [23], as well as in the one of Multifractal Theory of Motion [24,25], supposing that any polymer–drug system is assimilated both structurally and functionally to a multifractal object, the said dynamics can be described through motions of the polymer–drug structural units, dependent on the chosen scale resolution, on continuous and non-differentiable curves (multifractal curves). Such an assumption may be illustrated by considering the following scenario: between two successive interactions of the polymer–drug structural units, the trajectory of the polymer–drug structural unit is a straight line that becomes non-differentiable in the impact point. Considering that all interaction points form an uncountable set of points, it results that the trajectories of the polymer–drug structural units become continuous and non-differentiable (i.e., fractal curves). Clearly, the reality is much more complicated, taking into account both the diversity of the structural units which compose the polymer–drug system and the various interactions between them in the form of double interactions etc. Extrapolating the previous reasoning for any polymer–drug system, including our imine-chiotsan system, it results that it can be assimilated to a multifractal. The multifractal model is used here to characterize the dynamic of the atoms and molecule projecting their trajectories in the multifractal plane. Details on the fundamental links between the molecular or atomistic characteristics with the Multifractal Scale Relativity Theory can be found in [23,24,25].

All these considerations imply that in the description of the imine-chitosan dynamics, instead of “working” with a single variable (regardless of its nature, i.e., velocity, density, etc.) described by a strict non-differentiable function, it is possible to “work” only with approximations of this mathematical function, obtained by averaging them on different-scale resolutions. As a consequence, any variable purposed to describe the imino-chitosan dynamics will perform as the limit of a family of mathematical functions, this being non-differentiable for null scale resolutions and differentiable otherwise [23] (from a mathematical point of view, these variables can be explained through multifractal functions, i.e., functions dependent not only on spatial and temporal coordinates, but also on the scale resolution).

Since for a large temporal scale resolution with respect to the inverse of the highest Lyapunov exponent [28,29], the deterministic trajectories of any structural unit belonging to the polymer–drug system can be replaced by a collection of potential (“virtual”) trajectories, the concept of definite trajectory can be substituted by the one of probability density.

With all of the above considerations taken into account, the multifractality expressed through stochasticity, in the description of the dynamics of any polymer–drug system, becomes operational in the multifractal paradigm through the Multifractal Theory of Motion.

In this context, the authors’ study was directed to the modeling of the release of an antifungal aldehyde covalently bonded to chitosan by imine units. The in vitro release of aldehyde was investigated in an acidic medium of pH 4.2, characteristic to the vagina, which is highly susceptible to fungal infections. A mathematical model was created considering the imine-chitosan system as a multifractal object, and analyzing its dynamics in the framework of Scale Relativity Theory, using various operational procedures (Riccati-type gauges, isomorphisms of groups, joint invariant functions on groups, etc.). Finally, the theoretical model was validated by means of the experimental data.

## 2. Experiment

### 2.1. Materials

2-Formyl-phenyl-boronic acid, low molecular weight chitosan, and phosphate buffer solution were purchased from Aldrich (Sigma-Aldrich, Munchen, Germany) and used as received. The reagents used in antifungal measurements were purchased from Sigma-Aldrich (Sigma-Aldrich, Munchen, Germany) and used as received.

### 2.2. Synthesis of Imino-Chitosan Derivatives

Two chitosan derivatives with different aldehyde content were synthetized by reacting chitosan with 2-formyl-phenyl-boronic acid in a homogeneous medium, at 55 °C during 3 h, as described in reference [14]. Briefly, a 1% 2-formyl-phenyl-boronic acid in ethanol was slowly dropped into a 2% solution of chitosan in 0.7% acetic acid solution, under vigorous stirring at 55 °C for 3 h, when transparent yellowish hydrogels were obtained. Varying the ratio between the two reagents, two hydrogels containing 0.071% (coded C0.071) and 0.142% (coded C0.142) 2-formyl-phenyl-boronic were prepared.

### 2.3. Methods

The morphology of the hydrogels was investigated with a field emission Scanning Electron Microscope SEM EDAX Quanta 200 (FEI Company, U.S., Hillsboro, Oregon) at an accelerated electron energy of 20 KeV on small pieces of lyophilized hydrogel.

The supramolecular architecture of the hydrogels was assessed by polarized light microscopy on small hydrogel pieces placed between two lamellae, with an Olympus BH-2 polarized light microscope (Olympus BH-2 (Olympus company) Japan, Tokyo).

The swelling behavior of the two hydrogels was investigated by measuring the mass equilibrium swelling in an acidic medium of pH 4.2 [14].

The in vitro release of the antifungal aldehyde by shifting the imination equilibrium of the reagents was observed in an acidic medium of pH 4.2 similar to the vagina environment, by monitoring the absorbance of the aldehyde by UV-Vis and fitting it on a previously drawn calibration curve. Briefly, pieces of hydrogels were immersed in a buffer solution of pH 4.2. From time to time, 1 mL of supernatant was withdrawn and replaced with fresh buffer. The extracted supernatant was analyzed by UV-Vis spectroscopy and its concentration was found by fitting the absorbance on the calibration curve. Based on the obtained data, the release profile was drawn.

## 3. Empirical Data

**Figure 1:**
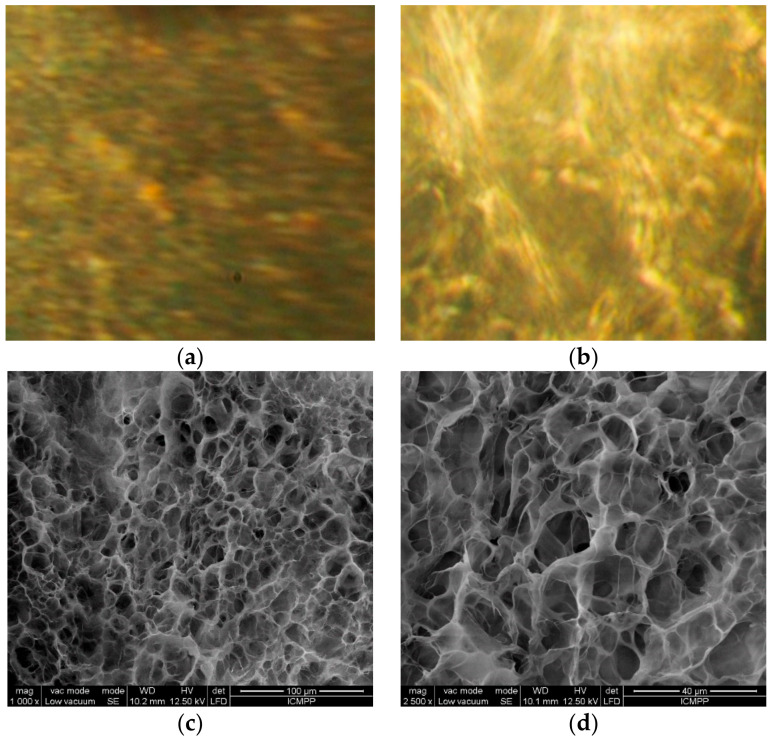
Representative (**a**,**b**) POM and (**c**,**d**) SEM images of the imino-chitosan hydrogels.

Two imino-chitosan derivatives were synthetized as hydrogels by reaction of chitosan with 2-formyl-phenyl-boronic acid through imination reaction. The hydrogelation occurred by two concurrent processes: (i) formation of imine units, and (ii) their supramolecular self-ordering into ordered layers [14]. This hydrogelation pathway was supported by the polarized light microscopy measurements, which displayed fine banded textures characteristic to the layered architectures, in line with the formation of ordered clusters of imino-chitosan, similar to smectic mesophases (Figure 1a,b) [30,31]. The hydrogel nature of the imino-chitosan derivatives was further demonstrated by scanning electron microscopy images, which showed a microporous morphology with pores of diameter in the 10–30 µm range, suitable for reaching sink conditions, during the release of antifungal aldehyde (Figure 1c,d).

**Figure 2:**
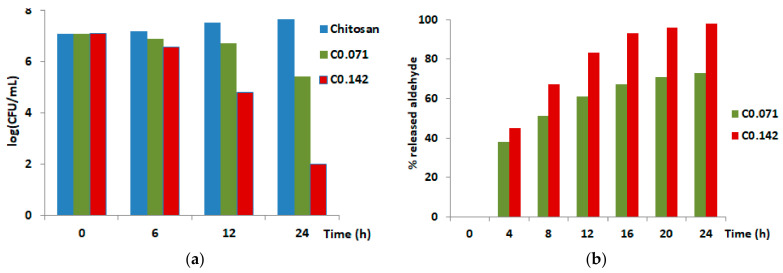
(**a**) Graphic representation of the in vitro fungicidal activity against planktonic yeast of *Candida albicans*; (**b**) The percent of antifungal aldehyde in vitro released during 24 h.

The hydrogels swelled in an acidic buffer of pH 4.2, reaching a mass equilibrium swelling of approximately 27, in line with a moisture medium inside the hydrogels that is favorable for the antifungal aldehyde release [32]. The in vitro investigation of the antifungal activity showed an excellent effect against *Candida albicans* and *Candida glabrata* strains [14], two virulent fungi accounting for systemic vulvovaginitis infections affecting women’s health [33]. While the chitosan reference sample only reduced the fungi growth, the two hydrogels showed a progressive killing of the strains, causing their almost complete extinction in the case of the C0.142 sample (Figure 2a). The decrease of the fungi population along with the increase of the amount of antifungal aldehyde in the samples indicated the aldehyde as a promoter of the antifungal activity. To confirm this, the in vitro release of aldehyde from the hydrogel samples has been monitored in a synthetic vagina-simulative medium, using the UV-Vis method. The release curves (Figure 2b) displayed a burst release in the first four hours, followed by a prolonged release. This profile shows that the hydrogel samples can assure the initial bolus dose of antifungal agent and further a constant dose necessary to kill the strains [34]. Comparing the in vitro antifungal effect with the in vitro release rate of the aldehyde, a close correlation can be clearly seen, meaning that the higher release rate of aldehyde from the C0.142 sample impacted a higher killing rate of the fungi. This means that the key role for the antifungal activity was played by the slow release of the antifungal aldehyde, mainly triggered by its reversible bonding on the chitosan under the pH stimulus. For a deeper understanding of this phenomenon, a mathematical model has been developed.

## 4. Theoretical Considerations

Taking into account the theoretical aspects presented in Section 1 by pursuing the transition approach from the scale space to the usual one, the imino-chitosan system release dynamics will imply the following:

(i) Aldehyde compound release dynamics in the scale space, dynamics which will be assimilated with the fungicidal activity;
(ii) Aldehyde compound release dynamics in the usual space, dynamics which will be assimilated with the antifungal aldehyde release;
(iii) Aldehyde compound release dynamics, associated with the transition from the scale space to the usual space, as a global release mechanism.

### 4.1. Aldehyde Compound Delivery Dynamics in Scale Space

Let a multifractal function $F\left(x\right)$ be considered with $x\in \left[a,b\right]$, which can be associated with any multifractal variable that describes drug delivery dynamics. Now, the sequence of the values of the variable x is considered:

(1) $$x_{a}=x_{0},\;x_{1}=x_{0}+\varepsilon ,\ldots ,\;x_{k}=x_{0}+k\varepsilon ,\;\ldots ,\;x_{n}=x_{0}+n\varepsilon =x_{b}$$

$F\left(x,\varepsilon \right)$ shall denote the fractured (broken) line connecting the points:

(2) $$F\left(x_{0}\right),\;\ldots ,\;F\left(x_{k}\right),\;\ldots ,\;F\left(x_{n}\right)$$

The broken line will be considered as an approximation that is different from the one used before. Let it be noted that $F\left(x,\varepsilon \right)$ is an ε-approximation scale. Now, the $\bar{\varepsilon }$-approximation scale $F\left(x,\bar{\varepsilon }\right)$ of the same function is considered. Since $F\left(x\right)$ is self-similar almost everywhere, if ε and $\bar{\varepsilon }$ are small enough, then the two approximations $F\left(x,\varepsilon \right)$ and $F\left(x,\bar{\varepsilon }\right)$ must lead to the same results when a multifractal drug release dynamics by approximations is studied. If the two cases are compared, then to an infinitesimal increase or decrease dε of ε, an increase or decrease $d\bar{\varepsilon }$ for $\bar{\varepsilon }$ corresponds, if the scale is dilated or contracted. In this case:

(3a) $$\frac{d\varepsilon }{\varepsilon }=\frac{d\bar{\varepsilon }}{\bar{\varepsilon }}$$

that is,

(3b) $$\frac{d\varepsilon }{\varepsilon }=d\mu$$

is the ratio of the scale ε+dε and dε must be preserved. Then, it is possible to consider the infinitesimal transformation of the scale as:

(4) $$\varepsilon^{\prime }=\varepsilon +d\varepsilon =\varepsilon +\varepsilon d\mu$$

By such a transformation, in the case of the function $F\left(x,\varepsilon \right)$, the following results:

(5) $$F\left(x,\varepsilon^{\prime }\right)=F\left(x,\varepsilon +\varepsilon d\mu \right)$$

Respectively, if a stop is made after the first approximation:

(6) $$F\left(x,\varepsilon^{\prime }\right)=F\left(x,\varepsilon \right)+\frac{\partial F}{\partial \varepsilon }\left(\varepsilon^{\prime }-\varepsilon \right)$$

that is:

(7) $$F\left(x,\varepsilon^{\prime }\right)=F\left(x,\varepsilon \right)+\frac{\partial F}{\partial \varepsilon }\varepsilon d\mu$$

Let it be considered that for an arbitrary but fixed $\varepsilon_{0}$:

(8) $$\frac{\partial \ln\left(\frac{\varepsilon }{\varepsilon_{0}}\right)}{\partial \varepsilon }=\frac{\partial \left(\ln\varepsilon -\ln\varepsilon_{0}\right)}{\partial \varepsilon }=\frac{1}{\varepsilon }$$

Thus, Equation $\left(7\right)$ becomes:

(9) $$F\left(x,\varepsilon^{\prime }\right)=F\left(x,\varepsilon \right)+\frac{\partial F\left(x,\varepsilon \right)}{\partial \ln\left(\frac{\varepsilon }{\varepsilon_{0}}\right)}d\mu$$

Finally:

(10) $$F\left(x,\varepsilon^{\prime }\right)=\left(1+\frac{\partial }{\partial \ln\left(\frac{\varepsilon }{\varepsilon_{0}}\right)}d\mu \right)F\left(x,\varepsilon \right)$$

The operator:

(11) $$\hat{D}=\frac{\partial }{\partial \ln\left(\frac{\varepsilon }{\varepsilon_{0}}\right)}$$

is a dilation or contraction operator.

This is the well-known form of the infinitesimal dilation operator, obtained above through the Gell–Mann–Levy method, which allows the finding of the currents corresponding for a given symmetry [35].

This clearly shows that the natural variable for the resolution is $\ln\left(\frac{\varepsilon }{\varepsilon_{0}}\right)$ and that the expected new differential equations involve quantities like $\frac{\partial F\left(x,\varepsilon \right)}{\partial \ln\left(\frac{\varepsilon }{\varepsilon_{0}}\right)}$.

In the previous context,

(12) $$\frac{\partial F\left(x,\varepsilon \right)}{\partial \ln\left(\frac{\varepsilon }{\varepsilon_{0}}\right)}=0$$

corresponds to the scale symmetry invariance, while the relation

(13) $$\frac{\partial F\left(x,\varepsilon \right)}{\partial \ln\left(\frac{\varepsilon }{\varepsilon_{0}}\right)}\neq 0$$

corresponds to the scale symmetry breaking. Since various problems related to complex system dynamics can be reduced to a Riccati-type gauge [15,16,17,28,29], in (13) such a gauge will be used, in the form of a Riccati-type equation:

(14) $$\frac{dw}{ds}-\frac{w^{2}}{M}+\frac{2R}{M}w-K=0$$

where:

(15) $$F\left(x,\varepsilon \right)\equiv w\left(s\right),\;s\equiv \ln\left(\frac{\varepsilon }{\varepsilon_{0}}\right)$$

and *M*, *R*, and *K* are constants of multifractal type introduced through external constrictions. Since the roots of the polynomial

(16) $$P\left(w\right)=\frac{w^{2}}{M}+\frac{2R}{M}w-K$$

can be written in the form

(17) $$w_{0}=R+iM\Omega ,{\bar{w}}_{0}=R-iM\Omega ,\;\Omega^{2}=\frac{K}{M}-{\left(\frac{R}{M}\right)}^{2}$$

the change of variable:

(18) $$z=\frac{w-w_{0}}{w-{\bar{w}}_{0}}$$

transforms (14) into:

(19) $$\dot{z}=2i\Omega z$$

of the solution:

(20) $$z\left(\tau \right)=z\left(0\right)e^{2i\Omega s}$$

**Figure 3:**
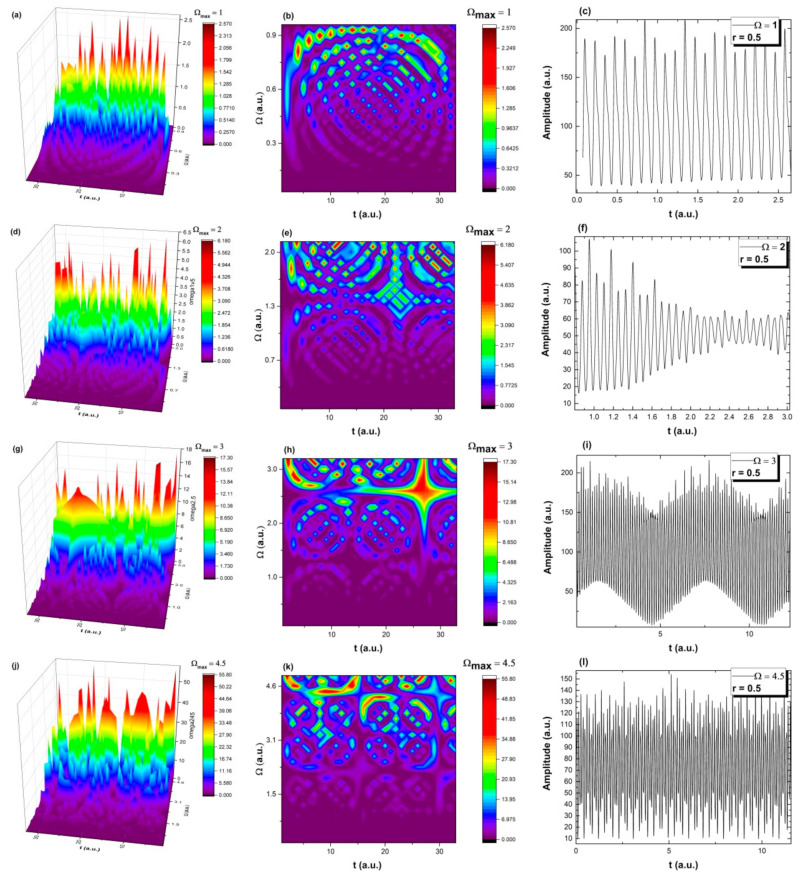
Transition scenarios in the aldehyde compound release dynamics and mimed as various modes of scale symmetry breaking (3D, contour plot and time series representation for *s* ≡t: period doubling (**a**–**c**), damped oscillation region (**d**–**f**), signal modulation (**g**–**i**) and chaotic behavior (**j**–**l**)).

As such, if the initial condition $z\left(0\right)$ is conveniently expressed, and then it is possible to construct the general solution of (14), by writing the transformation (18) in the form:

(21) $$w_{0}=\frac{w_{0}+re^{2i\Omega s}{\bar{w}}_{0}}{1+re^{2i\Omega s}}$$

where r is an integration constant of multifractal type. Using (17) it is possible to write this solution in real terms in the form:

(22) $$z=R+M\Omega \left\{\frac{2r\sin\left[2\Omega s\right]}{1+r^{2}+2r\cos\left[2\Omega s\right]}+i\frac{1-r^{2}}{1+r^{2}+2r\cos\left[2\Omega s\right]}\right\}$$

which can highlight various modes of scale symmetry breaking. These various modes are presented in Figure 3a–l. In the present context, it results that the natural transition in the aldehyde compound release dynamics is to evolve from a normal period doubling state towards damped oscillating and strong modulated dynamics. The imino-chitosan system never reaches a chaotic state, but it permanently evolves towards that state. There is a periodicity to the whole series of transitions and the imino-chitosan system evolves through period doubling, damped oscillations even reaching in some cases an intermittence state, but it never reaches a pure chaotic state. The evolution of the imino-chitosan system sees a “jump” into a period doubling oscillation state and the transition resumes towards a quasi-chaotic state. These dynamics are characteristic for short times and small distances, and are not usually seen for long time measurements. Such dynamics are true for a specific part of the release scenario being seen usually at the release interface and are strongly related with the polymeric structure and the multifractality of the matrix. The impossibility of reaching a chaotic state means that for a wide range of imino-chitosn systems, the release can be controlled to a high degree and there is no possibility for chaotic release and possible damage.

To better highlight this important stability property of the imino-chiotosan system, a study on the bifurcation map was attempted. It is observed that the imino-chitosan system starts from a steady state (double period state) and evolves towards pseudo-chaotic states ($\Omega_{max}=2,\;2.5,\;3\ldots$) but it never reaches that state. Here, the pseudo-chaos is defined by a high density of oscillations frequencies on which the imino-chitosan system oscillates simultaneously. This could be a measure of aldehyde release processes stability at a small space-time scale. Each train of scenarios follows the same path presented in Figure 3: double period oscillations, damped, modulated and quasi-chaotic oscillation, without ever reaching chaos. This means that at a microscale, the release could be different from the traces seen experimentally. The continuous increase in the amplitude of the oscillation reflects well the increase in released aldehyde compound mass as time evolves.

Let the implementation of the multifractal model at large scales be explored further, by introducing the following notation:

(23) r=cothμ

Then, (22) becomes:

(24) z=R+MΩh

with

(25) $$h=-i\frac{\cosh\mu +re^{2i\Omega s}\sinh\mu }{\cosh\mu +re^{2i\Omega s}\sinh\mu }$$

The significance of this parameter will be addressed later when aldehyde compound release dynamics will be analyzed. For the moment, it will be noted that the previous transition scenarios in the aldehyde compound release dynamics mimed as various modes of the scale symmetry breaking can be interpreted as phase self-modulation processes at various scale resolutions.

Let it be noted that the Ricatti-type differential Equation (14), rewritten in the notations:

M=2μ,  R=±c,  KM=−d

in the form:

(26) $$2\mu \frac{dw}{ds}=w^{2}\pm 2cu-d$$

admits, with the restriction $d+c^{2}=-KM+R^{2}>0$, the bounded solution

(27) $$s=-\frac{2\mu }{{\left(d+c^{2}\right)}^{\frac{1}{2}}}\tanh^{-1}\left[\frac{w\pm c}{{\left(d+c^{2}\right)}^{\frac{1}{2}}}\right]=-\frac{M}{{\left(-KM+R^{2}\right)}^{\frac{1}{2}}}\tanh^{-1}\left[\frac{w\pm R}{{\left(-KM+R^{2}\right)}^{\frac{1}{2}}}\right]$$

By setting:

(28) $$A={\left(d+c^{2}\right)}^{\frac{1}{2}}={\left(-KM+R^{2}\right)}^{\frac{1}{2}}$$

Then

(29) $$w\left(s\right)=c\pm A\tanh\left(\frac{As}{2\mu }\right)=R\pm {\left(-KM+R^{2}\right)}^{\frac{1}{2}}\tanh\left[\frac{{\left(-KM+R^{2}\right)}^{\frac{1}{2}}s}{M}\right]$$

**Figure 4:**
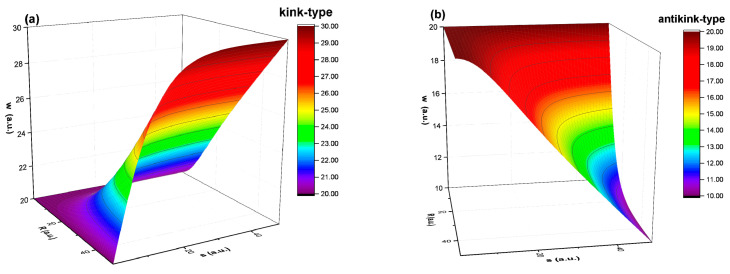
The multifractal kink (**a**) and antikink aldehyde (**b**) release modes.

Thus, there are specified behaviors of both multifractal kink type (+) as well as multifractal antikink type (−) in the drug release dynamics. For details on the standard kink and antikink solution see [28,29]. In Figure 4a,b, the multifractal kink and antikink aldehyde compound release modes of the imino-chitosan system are presented. The kink and antikink solutions found here describe two facets of the same problem. In the fractal paradigm, the aldehyde compound release processes can be best defined by the kink-type behavior. The simultaneous existence of both types of solution means that in the multifractal representation of the aldehyde release phenomena of the imino-chitosan system, ripples of the localized chemical interaction between the aldehyde compound and the medium can be present. For the case presented in this article, the decrease of the fungi population is reflected by the antikink type solution. Let it be noted that both solutions may exist at the same time, each contributing to create the complete image of the aldehyde release in the context of antifungal application.

Moreover, if the Ricatti-type differential Equation (14) is rewritten in the notations:

(30) $$M=-\frac{1}{f},\;\;R=\frac{1}{2},\;\;K=0$$

It will form logistic-type equations:

(31) $$\frac{dw}{ds}=fw\left(1-w\right)$$

**Figure 5:**
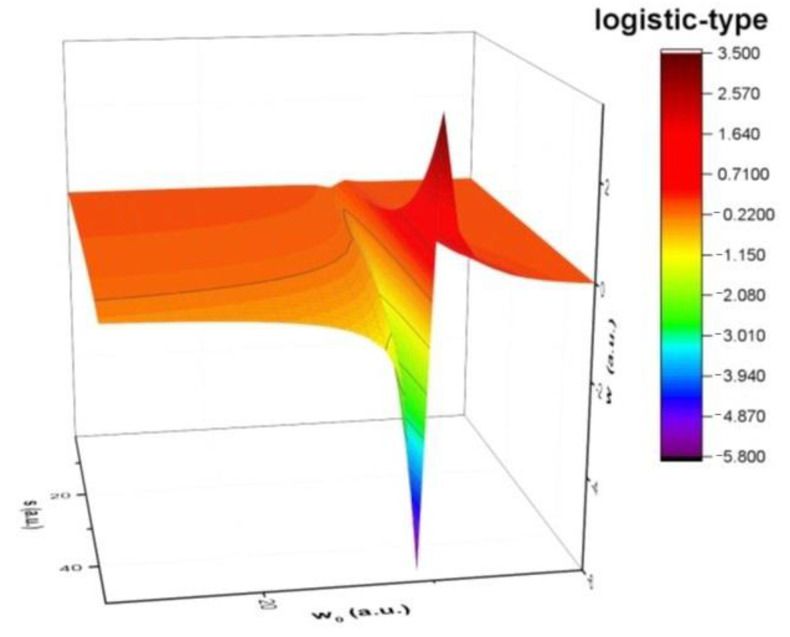
The logistic-type dependence of aldehyde release dynamics.

Its linearization by means of multiplication with $\frac{1}{w^{2}}$, i.e.,

(32) $$\frac{dn}{ds}=fn\left(1-n\right),\;\;n=\frac{1}{w}$$

implies, by means of integration, the solution:

(33) $$w=\frac{1}{1-\left(1-\frac{1}{w_{0}}\right)exp\left[-fs\right]}$$

where $w_{0}$ is an integration constant of multifractal type. Thus, the increase $\left(w\right)$ of aldehyde release dynamics of the imino-chitosan system is restricted by the self-interaction effects $\left(w^{2}\right)$ (the “finite world” effect). In Figure 5 the logistic-type dependence of aldehyde compound release dynamics from the imino-chitosan system are presented. It can be seen that around a fixed value for *w_0_* it is possible to observe the complete representation of the process. As the system reaches this optimal value, the aldehyde compound release is enhanced and reaches a maximum at *w*_0_ − Δ*w*. It can be seen that there is a negative contribution at *w_0_* + Δ*w* following the increase in aldehyde compound release, which means the medium changes (i.e., the fungal strains are killed changing the fractality of the background media in which the aldehyde compound are released). The symmetry of the solution highlights the synchronicity of the two phenomena and the coupling between them.

### 4.2. Aldehyde Compound Delivery Dynamics in the Usual Space

Considering the important role of the Riccati-type gauge in the dynamic analysis of drug release scenario in the space scale, in the following, such an analysis is extended for the aldehyde compound release dynamics in the usual space. Such an approach can be implemented through a Schrödinger equation of multifractal type:

(34) $$\lambda^{2}{\left(dt\right)}^{\left[\frac{4}{f\left(\alpha \right)}\right]-2}\partial^{l}\partial_{l}\Psi +i\lambda {\left(dt\right)}^{\left[\frac{2}{f\left(\alpha \right)}\right]-1}\partial_{t}\Psi =0$$

where:

(35) $$\partial_{t}=\frac{\partial }{\partial_{t}},\;\;\partial_{l}=\frac{\partial }{\partial X^{l}},\;\;\partial_{l}\partial^{l}=\frac{\partial }{\partial X^{l}}\left(\frac{\partial }{\partial X^{l}}\right)$$

based on a special invariance of the above equation, through the transformations [36]:

(36) $$X^{\prime }=\frac{X}{\gamma t+\delta },\;\;t^{\prime }=\frac{\alpha t+\beta }{\gamma t+\delta }$$

where α, β, γ, and δ are real elements.

In the above relation, ψ is the multifractal state function, $X^{l}$ with l=1,2,3 are the multifractal spatial coordinates, t is a non-multifractal temporal coordinate having the affine parameter role on the movement curves, dt is the scale resolution, λ is a coefficient associated to the multifractal-non-multifractal transition, $f\left(\alpha \right)$ is the singularity spectrum of order α, and α is the singularity index through which the fractal dimension $D_{F}$ is specified (for $D_{F}$ it is possible to use use any definitions—Kolmogorov fractal dimension, Hausdorff–Besikovich fractal dimension, etc. [37]; it is regularly found that $D_{F}$ < 2 for correlative processes and $D_{F}$ > 2 for non-correlative processes). From such a perspective, through $f\left(\alpha \right)$ it is possible to identify not only the aldehyde compound release volumes that are characterized by a certain fractal dimension (i.e., the case of monofractal drug release dynamics) but also the aldehyde compound release quantity for which the fractal dimension is situated in an interval of values (i.e., the case of multifractal drug release dynamics). Moreover, for the same $f\left(\alpha \right)$, it is possible to identify classes of universality in the aldehyde compound release dynamics laws, even when regular or strange attractors have various aspects [28,29].

Let it be observed that the transformation (36b) represents the homographic action of a matrix:

(37) $$\hat{\alpha }=\left(\begin{array}{cc} \alpha & \beta \\ \gamma & \delta \end{array}\right)$$

In such a context, the aldehyde release dynamics analysis in usual space is reduced to the obtainment of a relation between the matrix ensemble $\hat{\alpha }$ and an ensemble of t values through which t’ remains constant. Geometrically, this implies the searching of the ensemble of points (α, β, γ, δ), univocally corresponding to the values of the parameter *t*. Using (36b), the solution of the problem is reduced to a Riccati-type gauge in the form:

(38) $$dt+\omega_{1}t^{2}+\omega_{2}t+\omega_{3}=0$$

where the following notations are used [36]:

(39) $$\omega_{1}=\frac{\gamma d\alpha -\alpha d\gamma }{\Delta },\omega_{2}=\frac{\delta d\alpha -\alpha d\delta +\gamma d\beta -\beta d\gamma }{\Delta },\omega_{3}=\frac{\delta d\beta -\beta d\delta }{\Delta }$$

with

(40) Δ=α δ− γβ

It is easy to verify the fact that the metric:

(41) $$ds^{2}=\frac{{\left(\delta d\alpha +\alpha d\delta -\gamma d\beta -\beta d\gamma \right)}^{2}}{4\Delta^{2}}-\frac{d\alpha d\delta -d\beta d\gamma }{\Delta }$$

is in a direct relation with the discriminant from of the quadratic polynom (40):

(42) $$ds^{2}=\frac{1}{4}\left(\omega_{2}^{2}-4\omega_{1}\omega_{3}\right)$$

The three differentiable 1-forms from (39) completely define the coframe in every point of the absolute space. This coframe allows the translation of all geometric properties of the absolute space in the algebraic properties linked to (38).

The simplest property refers to dynamics on the metric geodesics, which can be correlated directly into statistical properties and from here to the multi-fractalization through stochasticization. In this case, the 1-forms $\omega_{1},\;\omega_{2},\;\omega_{3}$ are differentiable in the same parameters, i.e.,:

(43) $$\omega_{1}=a_{1}d\tau ,\;\omega_{2}=2a_{2}d\tau ,\omega_{3}=a_{3}d\tau$$

Along this geodesic, (38) becomes a Riccati-type equation:

(44) $$\frac{dt}{d\tau }=a_{1}t^{2}+2a_{2}t+a_{3}$$

with $a_{1},a_{2},\;a_{3}$ being constants that characterize certains geodesics from the family. From here, mathematical procedures can be implemented to obtain solutions similar to the ones presented in Section 4.2.

### 4.3. Aldehyde Compound Release Transitions from the Space Scale to the Usual One

Since any descriptions of aldehyde compound release dynamics are calibrated to Riccati-type equations, it implies algebraic structures of the SL(2R) type [29]. It results that the algebraic structure associated with the release dynamics in the scale space has to be isomorphic with the algebraic structure associated with the release dynamics in the usual space. Therefore, through the theorem of Stoka, the idea is to establish the joint invariant function that will also be the base of the aldehyde compound release transition from the space scale to the usual one. Thus, if the differential operators of the algebraic structure of SL(2R)-type associated with the scale space release dynamics are:

(45) $${\hat{A}}_{1}=\frac{\partial }{\partial h}+\frac{\partial }{\partial \bar{h}},\;{\hat{A}}_{2}=h\frac{\partial }{\partial h}+\bar{h}\frac{\partial }{\partial \bar{h}},\;\;{\hat{A}}_{3}=h^{2}\frac{\partial }{\partial h}+{\bar{h}}^{2}\frac{\partial }{\partial \bar{h}}+\left(h-\bar{h}\right)k\frac{\partial }{\partial k}$$

with the structure:

(46) $$\left[{\hat{A}}_{1},{\hat{A}}_{2}\right]={\hat{A}}_{1},\;\;\left[\;{\hat{A}}_{2},\;{\hat{A}}_{3}\right]=\;{\hat{A}}_{3},\;\;\left[\;{\hat{A}}_{3},{\hat{A}}_{1}\right]=-2{\hat{A}}_{2}$$

and the differentiable operators of the algebraic structure of SL(2R)-type associated to usual space release dynamics:

(47) $${\hat{B}}_{1}=\frac{\partial }{\partial z}+\frac{\partial }{\partial \bar{z}},\;{\hat{B}}_{2}=z\frac{\partial }{\partial z}+\bar{z}\frac{\partial }{\partial \bar{z}},\;\;{\hat{B}}_{3}=z^{2}\frac{\partial }{\partial z}+{\bar{z}}^{2}\frac{\partial }{\partial \bar{z}}$$

with the structure:

(48) $$\left[{\hat{B}}_{1},{\hat{A}}_{2}\right]={\hat{B}}_{1},\;\;\left[\;{\hat{B}}_{2},\;{\hat{B}}_{3}\right]=\;{\hat{B}}_{3},\;\;\left[\;{\hat{B}}_{3},{\hat{B}}_{1}\right]=-2{\hat{B}}_{2}$$

Then Stoka’s system becomes [34]:

(49) $${\hat{A}}_{i}f+{\hat{B}}_{i}f=0,\;i=1,2,3,\;f=f\left(z,\bar{z};h,\bar{h},k\right)$$

The general solution is a function based on the following algebraic functions:

(50a,b) $$\frac{h-z}{h-\overset{=}{z}}:\frac{\bar{h}-z}{\bar{h}-\overset{=}{z}}\equiv \rho^{2},\;\frac{h-z}{\bar{h}-z}k$$

Any joint invariant function is here a regular function of (50a,b). In (45) and (47),$\;\bar{h}$ is the complex conjugate of *h*,$\;\bar{z}$ is the complex conjugate of *z*, and *k* is a unidimensional factor.

For the case in which the joint invariant function is dependent online on (51a) with $\rho^{2}>0$, then for ρ=tanhμ, *z* is by (50a) related to *h* through the linear relation:

(51) $$z=u+vh_{0}$$

where h=u+iv is taken, provided $h_{0}$ is given by (25).

**Figure 6:**
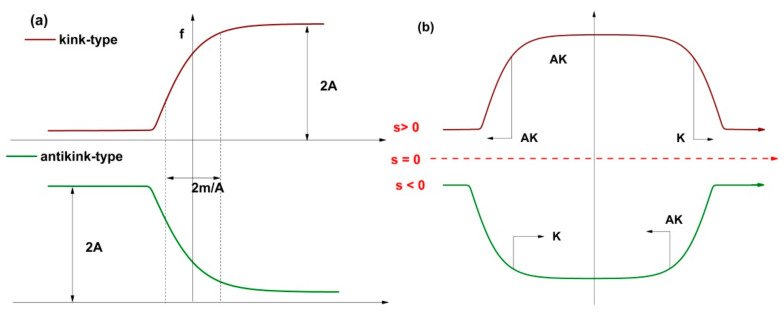
The dynamics of the kink–antikink pairs of multifractal type (**a**,**b**).

This isomorphism is responsible for the aldehyde compound release transitions between the scale space and the usual one. As such, aldehyde release dynamics can be interpreted through a joint invariant function of two isomorphic groups of SL(2R)-type, as drug release dynamics in the form of kink–antikink pairs’ dynamics of a multifractal type. The drug release dynamics through kink–antikink pairs of a multifractal type are presented in Figure 6.

**Figure 7:**
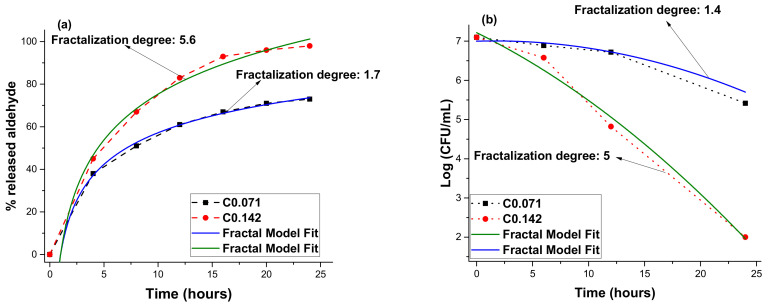
Fractal model fit on the empirical data for aldehyde release (**a**) and fungal activity (**b**).

In Figure 7, the calibration of the experimental data relative to the proposed theoretical model is presented. A good consistency of data between the theoretical model and the experimental data can be observed. From Figure 2 it can be observed that the imino-chitosan derivatives have a porous quasi-fractal structure. This morphology is expected to affect the drug release dynamics as well. A higher fractality on the surface will be reflected in the fractality of the system. As with any fractal system, the scale transition from polymer to the in vitro system is dependent on the fractality of the release process. Therefore, the C0.071 system will be described by a higher fractality degree and a faster release dynamic coupled with enhanced fungal activity. The C0.142 case is described according to the authors’ proposed model, by a fractality degree of 1.7, which is a factor of 3.2 lower than the C0.071 system. The lower fractality will induce a lower release rate and lower fungal activity. The good correlation between the fractality degree of the system and the drug-release behavior can have a potentially large impact on tailoring new polymer–drug configurations and improving their properties.

## 5. Conclusions

The paper reports for the first time a theoretical model for the release of a bioactive compounds covalently bonded to a matrix, i.e., on an imine-chitosan system obtained by covalent bonding of an antifungal aldehyde to the chitosan via reversible imine linkages. The premises for creating this model were the fungicidal activity on Candida strains of the system and the in vitro release curves of the antifungal aldehyde. Their good agreement proved that the reversible bonding of aldehyde triggers its controlled release. Starting from these premises, the model has been created considering the imino-chitosan system as a multifractal object—a mathematical model for the release of bioactive aldehyde due to the reversible imine linkages. The release dynamics were analyzed in the framework of the Scale Relativity Theory, and more precisely in the form of the Multifractal Theory of Motion. Through Riccati-type gauges, two synchronous dynamics, one in the scale space, associated with the fungicidal activity, and the other in the usual space, associated with the antifungal aldehyde release, become operational. Their synchronicity, reducible to the isomorphism of two $\mathrm{SL}\left(2R\right)$-type groups, implies, by means of its joint invariant functions, aldehyde compound release dynamics in the form of kink–antikink pairs’ dynamics of a multifractal type. The synchronicity of the two phenomena and the coupling between them was highlighted by the symmetry of the solutions of the two operational procedures. The theoretical model has been validated through its good consistency with the experimental data.

## Data Availability

The data will be available upon request by the corresponding authors.

## Sample Availability

Samples of the compounds are not available from the authors.
